# Flavonoids and fibrate modulate apoE4-induced processing of amyloid precursor protein in neuroblastoma cells

**DOI:** 10.3389/fnins.2023.1245895

**Published:** 2023-12-22

**Authors:** Viralkumar Davra, Kenza E. Benzeroual

**Affiliations:** Department of Pharmaceutical Sciences, Arnold and Marie Schwartz College of Pharmacy and Health Sciences, Long Island University, Brooklyn, NY, United States

**Keywords:** flavonoids, diosmetin, naringenin, fibrates, APP, amyloid β, apoE4, neuronal cells

## Abstract

**Introduction:**

Apolipoprotein (apo) E4, being a major genetic risk factor for Alzheimer’s disease (AD), is actively involved in the proteolytic processing of amyloid precursor protein (APP) to amyloid β (Aβ) peptide, the principle constituent of amyloid plaques in Alzheimer Disease (AD) patients. ApoE4 is believed to affect APP processing through intracellular cholesterol homeostasis, whereas lowering the cholesterol level by pharmacological agents has been suggested to reduce Aβ production. This study has investigated the effects of hypolipidemic agents fenofibrate, and the flavonoids–naringenin and diosmetin–on apoE4-induced APP processing in rat neuroblastoma cells stably transfected with human wild-type APP 695 (B103-hAPP695wt).

**Results:**

B103-hAPP695wt cells were pretreated with different doses of flavonoids and fenofibrate for 1 h prior to apoE4 exposure for 24 h. ApoE4-induced production of intra- and extracellular Aβ peptides has been reduced with fenofibrate, naringenin, and diosmetin treatments. Pretreatment with diosmetin has significantly reduced apoE4-induced full-length APP (fl- APP) expression, whereas naringenin and fenofibrate had no effect on it. In addition, the increase in the apoE4-induced secretion of sAPPtotal and sAPPα has been dose-dependently reduced with drug pretreatment. On the other hand, the decrease in the expression of both APP-carboxy terminal fragments (CTF)-α and –β (generated by the α- or β-secretase cleavage of APP) by apoE4 was dose-dependently increased in cells pretreated with fenofibrate and naringenin but not diosmetin.

**Conclusion:**

Thus, we suggest that fenofibrate, naringenin, and diosmetin treatments can reduce apoE4- induced Aβ production by distinct mechanisms that may prove useful in developing drugs for AD patients.

## 1 Introduction

Alzheimer’s disease (AD) is the most common irreversible neurodegenerative dementia (Lane et al., 2018). One of the neuropathological hallmarks of the disease includes extracellular deposition of senile neuritic plaques composed of Amyloid-β (Aβ) peptides (Masters et al., 1985). Aβ peptide accumulation and aggregation leads to progressive neuronal, synaptic, and memory loss (Mattson, 2004).

Aβ peptide is generated from the amyloid precursor protein (APP), which is a central protein involved in AD pathology (Dyrks et al., 1988). The APP has three common isoforms: APP695, APP750, and APP 771. The APP695 is predominantly expressed in neurons in the central nervous system (CNS) (Zhang et al., 2011). The APP undergoes sequential proteolytic cleavage by the coordinated actions of the membrane-embedded α-, β-, and γ-secretase enzymes. APP is first cleaved at the aminoterminal by β-secretase (also known as β-site APP cleaving enzyme (BACE1)) to generate a large soluble APP-β (sAPPβ) as well as a shorter membrane-retained carboxy-terminal fragment-β (CTFβ) (Vassar et al., 1999). The resultant CTFβ is cleaved by a presenilin (PS1)-dependent γ-secretase to generate a 4-kDa soluble Aβ fragment released in the extracellular space (Haass, 2004) and the APP intracellular domain (AICD) in the cytosol. Aβ peptide can vary in length and the most common isoforms are Aβ38, Aβ40, and Aβ42 (Haass et al., 1992; Iwatsubo et al., 1994). Although the Aβ40 is highly abundant (~90% of total Aβ), Aβ42 is considered more toxic (Umeda et al., 2011). In an alternative non-amyloidogenic pathway, α-secretase cleaves APP within the Aβ region, leading to the production of a large secretory soluble amyloid precursor protein-α (sAPPα) and a membrane-bound carboxy-terminal fragment-α (CTFα) (Vingtdeux and Marambaud, 2012). Cleavage of the CTFα by the γ-secretase generates non-toxic p3 peptides and AICD (Haass et al., 1993). While α-secretase cleavage of APP seems to take place outside of lipid rafts (Parr-Sturgess et al., 2010), the processing by β- and γ-secretases is lipid raft-associated (Vetrivel et al., 2004). Indeed, studies reported that the proteolytic processing of APP is influenced by the lipid composition of the cell membrane (Beel et al., 2008; Grimm et al., 2012) and that it has also been found to be altered in AD post-mortem brains (Grimm et al., 2011; Naudí et al., 2015). In addition, a recent large genome-wide association (GWA) meta-analysis of clinically diagnosed late-onset Alzheimer’s disease (LOAD) that was conducted to identify new risk loci for LOAD also revealed that the pathway analysis implicates lipid metabolism and APP metabolism, showing that genetic variants affecting APP and Aβ processing are associated not only with early-onset autosomal dominant Alzheimer’s disease (EOAD) but also with LOAD (Kunkle et al., 2019).

To date, the only gene consistently found to be associated with LOAD is the *apolipoprotein E* (*apoE*) gene (Corder et al., 1993; Sims et al., 2020). ApoE protein is abundantly produced in the brain and mainly from astrocytes with significant function in CNS integrity and neuronal remodeling (Boschert et al., 1999). ApoE protein also plays a critical role in the transport and metabolism of cholesterol (Mahley, 2016), and cholesterol has been reported to increase Aβ production (Howland et al., 1998) by affecting α-, β-, and γ-secretase enzyme activity (Simons et al., 1998; Kojro et al., 2001). Of the three common polymorphic apolipoproteins (E2, E3, and E4), the apoE4 isoform has been considered the major genetic risk factor for LOAD (Czech et al., 1993; Serrano-Pozo et al., 2021). In total, 40% of all AD patients have at least one inherited apoE4 gene, while being homozygous for the apoE4 allele increases the risk of AD by 10-fold (Yu et al., 2014; Cuyvers and Sleegers, 2016). GWA studies identified genes involved in cholesterol metabolism or transport as AD susceptibility genes and suggested that apoE4 mechanisms may be lipid-related (Harold et al., 2009; Jones et al., 2011). While several studies suggested a strong link between cholesterol metabolism and the formation of Aβ peptides, the evidence regarding the role of lipid-lowering agents in reducing dementia risk has been mixed.

Fenofibrate is a widely used hypolipidemic drug with neuroprotective benefits probably through the modulation of the nuclear receptor peroxisome proliferator-activated receptor (PPAR) alpha (PPARα) (Sáez-Orellana et al., 2020). PPARα agonists activate the α-cleavage of APP (Corbett et al., 2015) but inhibit the β-secretase (BACE1) enzyme with no effect on the level of APP and Presenilin-1 (PS1) (Zhang et al., 2015). Conflicting studies have been reported for Fenofibrate. Fenofibrate can act as an inverse γ-secretase modulator (GSM) such that it favors Aβ peptide production and also inhibits its degradation independently of γ-secretase modulation (Kukar et al., 2005; Gamerdinger et al., 2007; Abdul-Hay et al., 2009). Others showed that fenofibrate increased the expression and activity of PPARα and reduced Aβ levels most likely via the phosphatidylinositol 3-kinase (PI3-K) pathway (Zhang et al., 2014; Assaf et al., 2020). To better understand the role of fenofibrate, a modulator of PPARα, in AD, we examined its ability to regulate apoE4-induced APP processing in neuronal cells.

Flavonoids are a class of phenolic compounds commonly present in plants with widely recognized biochemical and pharmacological actions. Naringenin has been reported to possess antihyperlipidemic activity by inhibiting the acyl-coenzyme A-cholesterol acyltransferase (ACAT) (Lee et al., 2001; Jeon et al., 2007), but its role in the modulation of APP processing into Aβ has not been investigated yet. Indeed, ACAT inhibitors have been extensively studied as Aβ modulators in neuronal and non-neuronal cells to attenuate Aβ production (Puglielli et al., 2001; Huttunen et al., 2009). Another flavonoid, diosmetin which is the aglycone portion of the flavonoid glycoside diosmin (Barreca et al., 2020) has multiple health benefits including anti-lipolytic activity (Lee et al., 2020; Garg et al., 2022). A recent study showed that diosmetin decreased APP upregulation and β-secretase (BACE1) expression, thus reducing Aβ production in advanced glycation end products (AGEs)-induced Alzheimer’s disease (AD)-like pathology in neuronal cells probably through the activation of the peroxisome proliferator-activated receptor-gamma (PPARγ) (PPARγ) pathway (Lai et al., 2022). While PPARs are expressed in brain cells, PPARγ is both expressed in neurons and astrocytes (Warden et al., 2016). PPARγ was previously demonstrated to also regulate Aβ production by controlling the expression of β-secretase (BACE1) gene (Wang et al., 2013). PPARγ is expected to provide a new therapeutic approach for the prevention of neurodegenerative diseases, including AD (Ding et al., 2020). Therefore, diosmetin, a vascular protectant, could be considered a potential candidate for novel anti-AD therapy. Indeed, vascular dementia is the second most prevalent type of dementia and is a significant risk factor for the development of AD and is more likely in patients homozygous for apoE4 (Li et al., 2011; Rohn, 2014).

This study investigated the effects of the lipid-lowering agents such as fenofibrate, and flavonoids–naringenin and diosmetin–on apoE4-induced APP processing into Aβ peptides in B103-hAPP695wt cells. We investigated the expression of full-length amyloid precursor protein (fl-APP). CTFα and CTFβ using the Western blot analysis of cellular protein lysate. The secretion of soluble APPs (sAPPtotal and sAPPα) was also measured by immunoblotting of the conditioned media. Intracellular and extracellular levels of Aβ were quantified using a quantitative ELISA. The expression of fl-APP mRNA was also measured using the Rt-PCR experiment. To this end, our results showed that fenofibrate, naringenin, and diosmetin have attenuated the apoE4-induced APP processing into Aβ peptides.

## 2 Materials and methods

### 2.1 Cell line and material

Rat neuroblastoma B103 cells stably expressing human wild-type APP 695 isoforms (B103hAPP695wt) were generously provided by Gladstone Institute of Neurological Disease (GIND), UCSF, *CA.* Human recombinant apoE4 (apoE4) was obtained from Bio-Vision Inc, Waltham, MA. The flavonoid, diosmetin, was obtained from Indofine Chemical Company, while naringenin and fenofibrate were obtained from Sigma Aldrich (St. Louis, MO). Rat monoclonal antibodies (APP, Amyloid β, and secondary antibodies) were from Santa Cruz biotechnologies (SCBT). Monoclonal Aβ (6E10) was obtained from Covance Inc, Waltham, MA. Tissue culture reagents were obtained from Sigma–Aldrich. ELISA kits for Aβ were purchased from Invitrogen.

### 2.2 Cell culture and drug treatment

Rat neuroblastoma B103 cells stably expressing human wild-type APP (hAPP695wt) (B103-hAPP695wt cells) were cultured in high glucose Dulbecco’s modified Eagle’s medium (DMEM) containing 400 μg/mL G418 disodium salt solution, 10% fetal bovine serum, 5% horse serum, 1% sodium pyruvate, and 1% penicillin–streptomycin in an incubator with a constant supply of 5% CO_2_ at 37°C. For differentiation, sub-confluent B103-hAPP695wt cells were washed twice with serum-free DMEM and incubated for 24 h in DMEM containing 1% N2 supplement (Ye et al., 2005).

**Figure 1:**
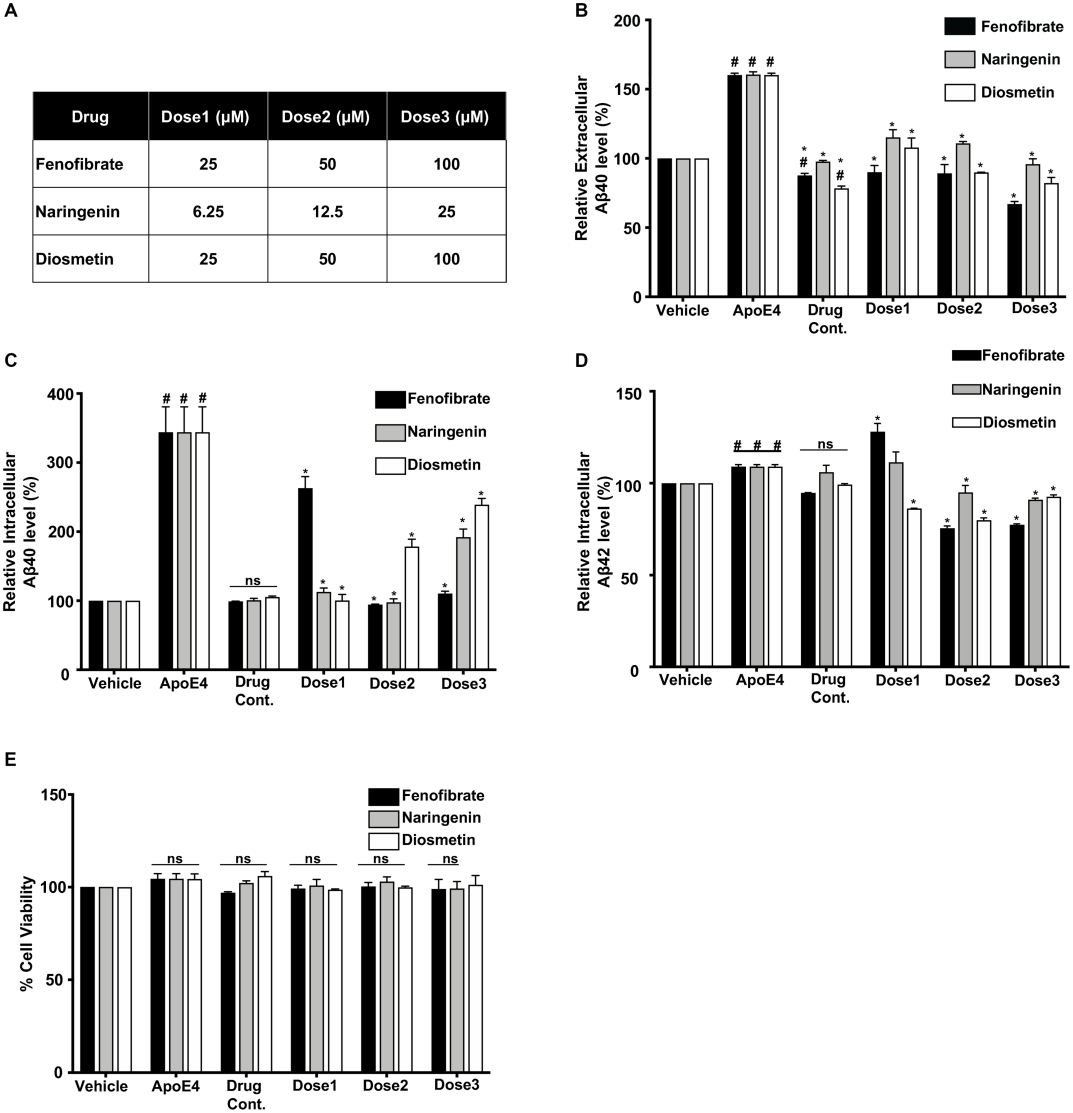
Comparative effect of different concentrations (Dose1, Dose2, and Dose3) of fenofibrate (25, 50, and 100 μM), naringenin (6.25, 12.5, and 25 μM), and diosmetin (25, 50, and 100 μM) **(A)** on the secretion of extracellular Aβ40 **(B)** and the production of intracellular Aβ 40 **(C)** and Aβ42 **(D)** as measured by ELISA, and cell viability **(E)** as measured by MTT assay, in apoE4- induced B103-hAPP695wt cells. Cells were treated with vehicle (0.1% DMSO), apoE4 (7.5 μg/mL), or drug control only (Dose3 (100 μM) fenofibrate, Dose3 (6.25 μM) naringenin, and Dose3 (100 μM) diosmetin) for 24 h. Pretreatment consisted of fenofibrate, naringenin, and diosmetin for 1 h prior to stimulation with apoE4 for 24 h. Quantitative evaluation for the secretion of extracellular Aβ and the production of intracellular Aβ was normalized against the respective cellular protein concentration of treated cells. For statistical evaluations, the two-way ANOVA analysis followed by Tukey’s range test was performed. Statistically significant differences (#*p* < 0.05) compared with vehicle; (*p < 0.05) compared with apoE4-treated cells only, not significantly different (ns) compared to vehicle. Data are expressed as percentage of protein expression compared with vehicle, and mean ± S.D. of at least three separate experiments.

Differentiated B103-hAPP695wt cells were treated as follows: control (no treatment), vehicle (0.1% DMSO), apoE4 (7.5 μg/mL), and drug control (fenofibrate at 100 μM, naringenin at 25 μM or diosmetin 100 μM) for 24 h. The highest dose (Dose3 under Figure 1A) used in this study was selected as a drug control dose to ensure that at the highest dose, the drug is not killing cells. For all other conditions, cells were pretreated with varying concentrations of fenofibrate (25, 50, and 100 μM), naringenin (6.25, 12.5, and 25 μM), and diosmetin (25, 50, and 100 μM) for 1 h (hr) prior to apoE4 (7.5 μg/mL) exposure for 24 h. All drugs were dissolved in DMSO, and the final concentration of DMSO in cell culture media was less than 0.1%. The differentiated B103-hAPP695wt cells were examined under the microscope after 24 h for changes in morphology and viability. B103-hAPP695wt cell viability was also determined using the trypan blue exclusion test and the MTT assay.

### 2.3 MTT cell viability assay

Cell viability was determined by 3-(4,5-dimethylthiazol- 2-yl)-2,5-diphenyltetrazolium bromide (MTT) reduction assay, which is based on the conversion of MTT to purple formazan crystals by mitochondrial succinate dehydrogenases. The cells were seeded in triplicate at the density of 25 × 10^3^ cells/well in a 96-well plate and treated as described previously. MTT solution (0.5 mg/mL) was then added to each well, and the plates were incubated at 37°C with a constant supply of 5% CO_2_ and 95% air-humidified environment for 4 h. The medium was discarded and 100 μL of sterile dimethyl sulphoxide (DMSO) was added to each well, and the plate was agitated on an orbital shaker for 15 min to dissolve the formazan crystals formed in intact cells. A microplate reader (Bio-Rad) was used to measure the optical density at 550 nm. Results were expressed as the percentage of reduced MTT, considering the optical density of cells treated with the vehicle as 100%.

### 2.4 Enzyme-linked immunosorbent assay

The levels of Aβ40 and Aβ42 in the conditioned medium (extracellular Aβ) and the cellular protein lysate (intracellular Aβ) were determined by ELISA using 6E10 as the capture antibody, and biotinylated IBR 5-139mAb for Aβ40 and 1–11-3R mAb for Aβ42 as the detection antibodies. Briefly, 96 wells of microtiter plates were coated with 100 μL of human Aβ40 or Aβ42 (6E10) antibody (0.5 or 0.25 μg/mL), diluted in carbonate–bicarbonate buffer, pH 9.6, and incubated at 4°C overnight. Plates were then washed with PBS containing 0.05% tween-20 (PBST) and blocked for 1 h with 1% BSA in PBST to avoid non-specific binding. The plates were washed again, and 100 μL of samples and standards (human Aβ, Bachem, CA) diluted in hexafluoroisopropanol was applied and incubated for 2 h at room temperature and at 4°C overnight. Aβ peptides were detected with biotinylated mAbs. After washing, neutravidin horseradish peroxidase conjugate was added followed by color development with the 3,3′,5.5′-tetramethylbenzidine substrate solution. The reaction was stopped by 1 M phosphoric acid, and the optical density was measured at 450 nm in a microELISA reader. In addition, commercial Aβ 40 and Aβ42 ELISA (Invitrogen) were also used in accordance with the manufacturer’s instructions. Equivalent volumes of conditioned medium or cellular lysate were loaded per well, and Aβ levels were normalized according to the respective cellular protein content. Commercial Aβ ELISA (Invitrogen) was also used in accordance with the manufacturer’s instructions. Equivalent volumes of conditioned medium or cellular lysate were loaded per well, and Aβ levels were normalized according to the respective cellular protein content. Consistent results were obtained with both ELISA methods.

### 2.5 Western blot analysis

Following drug treatment, the conditioned medium from each sample was harvested, and cellular proteins were extracted using RIPA lysis buffer. Protein concentration was measured using the Pierce BCA protein assay kit, and an equal amount of proteins were blotted using the Western blot assembly. The expression of secreted APP (sAPP) and cellular APP (FL-APP) was detected in the conditioned medium and cellular extract, respectively, on 8% polyacrylamide gel using human APP mAb 3E9 (1:1000) and 6E10 Ab (1:2000), with overnight incubation. For detection of CTFs, proteins were resolved on 16.5% Tris-tricine gel and probed using mouse monoclonal β-Amyloid (H-43) Ab (1:500). The nitrocellulose membranes were then probed with HRP conjugated anti-rat secondary antibody (1:2000). Protein expression was revealed using the enhanced chemiluminescence ECL system, and the images were captured using the gel documentation system, Gel-Logic 2,200 pro imager. β-actin (1,4,000) expression in respective blots was probed to ensure equal protein loading and protein expression was represented as a percentage of control.

### 2.6 Real-time reverse transcription – PCR analysis

Total RNA was isolated from sub-confluent treated B103-hAPP695wt cells using RNeasy Mini Kit according to the manufacturer’s instructions. The concentration of total RNA in a sample was determined using a Nanodrop instrument. Reverse transcription was performed on 1 μg of total RNA for cDNA (first strand DNA) synthesis using the Super Script VILO cDNA Synthesis Kit. Real-time PCR was performed in a 50-μl reaction volume containing 2 μL of cDNA, 1 μL of forward and reverse primers, and 25 μL of 2X SYBR Green JumpStart Taq Ready Mix (Sigma Aldrich, MO) using the iCycler real-time thermocycler (Bio-Rad Laboratories, Hercules, CA). The PCR cycle number that generated the first fluorescence signal above the threshold (CT) was determined. The generation of specific PCR products was confirmed by melting-curve analysis. The following primers were used: for human APP695, 5'-ATTCTTTTACGGCGGATGTG-3′ (forward) and 5'-CTTGACGTTCTGCCTCTTCC-3′ (reverse) and for rat β-Actin, 5'-GAGAGGGAAATCGTGCGTGAC-3′ (forward) and 5'-CATCTGCTGGAAGGTGGACA-3′ (reverse).

PCR conditions were initial denaturation at 94°C for 2 min, followed by 40 cycles at 94°C for 15 s and 60°C for 1 min. APP mRNA fold change relative to β-Actin mRNA was calculated using the qPCR delta–delta Ct method.

### 2.7 Statistical analysis

The results were presented as mean ± standard deviation (SD) of at least three independent experiments. Comparison between experimental groups was determined using analysis of variance (ANOVA). Specific pair-wise differences were determined using Tukey’s range test. Differences were considered statistically significant when value of *p* was <0.05.

## 3 Results

ApoE4 has been well documented to induce APP processing and Aβ production in *in vitro* experimental models of AD (Ye et al., 2005). To investigate the effect of cholesterol-modulating agents on apoE4-induced Aβ production, B103-hAPP695wt cells were treated with varying concentrations of fenofibrate (25, 50 and 100 μM), naringenin (6.25, 12.5, and 25 μM), and diosmetin (25, 50 and 100 μM) for 1 h prior to apoE4 (7.5 μg/mL) exposure for 24 h. After 24 h of treatment, cell lysates and conditioned media were harvested, and intracellular and extracellular Aβ levels were quantified using specific sandwich ELISAs. Immunoblot analysis was performed with the cell lysates for the detection of full-length APP (fl-APP) and CTFα and CTFβ. The conditioned media was also used for the detection of total soluble APP (sAPPtotal) and sAPPα. RNA extracted from treated B103-hAPP695wt cells was used to carry out the quantitative Rt-PCR experiment to investigate APP mRNA expression.

### 3.1 Effect of fenofibrate and flavonoids on apoE4-induced Aβ peptides secretion and production

To evaluate the effects of fenofibrate and flavonoids on apoE4-induced Aβ production, the levels of extracellular Aβ peptides were determined by measuring the secretion of human Aβ40 and Aβ42 in the conditioned medium using selective sandwich ELISAs. Overall, cells treated with drug controls showed no significant increase as compared to vehicle (DMSO) treated cells. Levels of Aβ in the conditioned medium of the B103-hAPP695wt cells treated with vehicle (DMSO) were considered as 100%. The treatment with 7.5 μg/mL apoE4 has significantly increased the secretion of Aβ40 by 0.6-fold, as compared to vehicle-treated cells (Figure 1B).

Pretreatment of cells with fenofibrate at 25, 50, and 100 μM has decreased apoE4-induced secretion of Aβ40 level in the conditioned medium by 0.70-, 0.71-, and 0.93-folds, respectively. Similarly, pretreatment of cells with naringenin at 6.25, 12.5, and 25 μM has decreased the secretion of Aβ 40 level by 0.45-, 0.49-, and 0.64-folds, while diosmetin at 25, 50, and 100 μM has decreased the secretion of Aβ40 level by 0.52-, 0.70-, and 0.78-folds respectively, as compared to apoE4- treated cells. All pre-treatments have reduced the secretion of Aβ 40 level dose-dependently and significantly as compared to apoE4-treated cells (Figure 1B). The protective effect was pronounced with fenofibrate followed by diosmetin then naringenin. The levels of extracellular Aβ42 were below the detection limit in cells pretreated with fenofibrate and flavonoids.

Levels of intracellular human Aβ40 and Aβ42 were determined in the cellular protein lysate using specific ELISAs (Figures 1C,D). Aβ levels in the vehicle (DMSO)-treated cells were considered as 100%. The production of intracellular Aβ40 in the apoE4-treated B103-hAPP695wt cells was increased significantly by 2.59-fold as compared to vehicle-treated cells. There were no significant changes observed in the intracellular Aβ production with drug controls as compared to vehicle. Similar to extracellular Aβ40, fenofibrate pretreatment has significantly decreased the apoE4-induced intracellular Aβ40 level by 0.96, 2.64-, and 2.48 folds at 25, 50, and 100 μM doses, respectively. The pretreatment with naringenin at 6.25-, 12.5, and 25 μM has significantly reduced the Aβ production by 2.46-, 2.61-, and 1.67-folds, respectively, whereas the pretreatment with diosmetin at 25, 50, and 100 μM has also significantly reduced the intracellular Aβ40 production by 2.58-, 1.80-, and 1.2-folds, respectively, as compared to apoE4-treated cells. Naringenin and diosmetin effects were more pronounced at the lowest dose compared to fenofibrate (Figure 1C). On the other hand, apoE4 treatment significantly increased the production of intracellular Aβ42 by 0.09-fold as compared to the vehicle cells (Figure 1D). While fenofibrate at the lowest concentration (25 μM) did not prevent apoE4-induced Aβ42 generation but rather showed an even higher level, pretreatment of cells with 50 and 100 μM has significantly decreased the production of intracellular Aβ42 levels by 0.33- and 0.32-folds, respectively, as compared to apoE4-treated cells. Similarly, pretreatment of cells with 12.5 and 25 μM naringenin also significantly decreased the apoE4-induced production of Aβ42 level by 0.14- and 0.18-folds, respectively. Diosmetin at 25, 50, and 100 μM has decreased the production of Aβ42 level by 0.22-, 0.29-, and 0.16-folds compared to apoE4-treated cells (Figure 1D). Overall, at the highest dose, the lowering effects on apoE4-induced intracellular Aβ40 and Aβ42 were more prominent with fenofibrate, followed by naringenin than diosmetin.

These results suggest that all drug treatments prevented the apoE4-induced APP processing into Aβ peptides. The decrease in the levels of Aβ in B103-hAPP695wt cells was not due to the apoE4- or drug-induced neurotoxicity as confirmed by the MTT cell viability assay (Figure 1E).

### 3.2 Effect of fenofibrate, naringenin, and diosmetin on apoE4-induced full-length APP protein and mRNA expression

To investigate the protective effect of fenofibrate and flavonoids on apoE4-induced Aβ production, we measured the expression of fl-APP as the Aβ peptide is the direct proteolytic product of fl-APP. The level of fl-APP was assessed in the cellular lysate using β-Amyloid (6E10) Ab, an antibody that recognizes APP (674–679 aa) at the C terminus, and APP mAb 3E9, a human antibody that recognizes APP (18–38 aa) at the N terminal.

**Figure 2:**
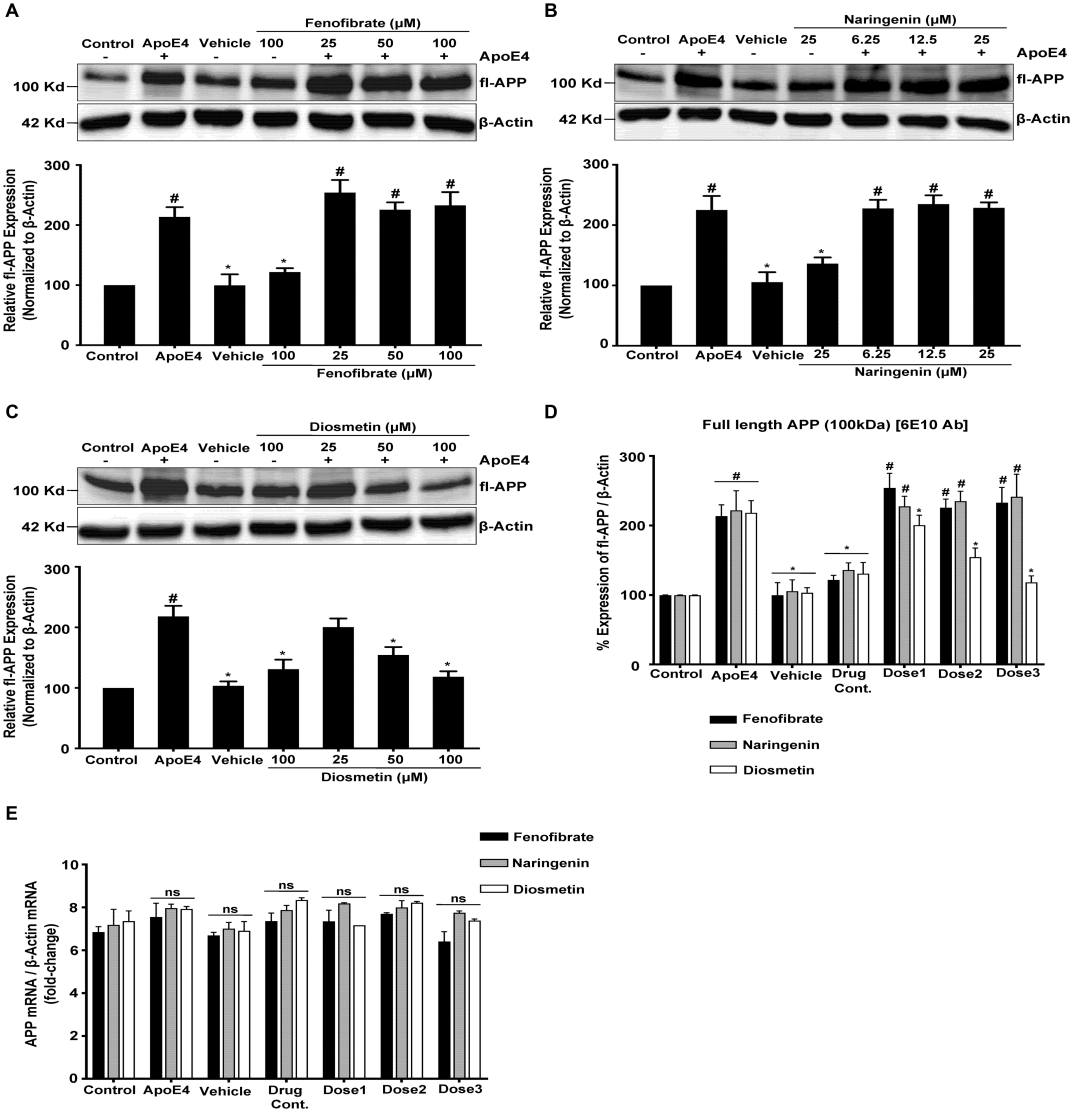
Effect of fenofibrate **(A)**, naringenin **(B)**, and diosmetin **(C)** on fl-APP expression as measured by the Western blot analysis using mAB 3E9 and 6E10 Ab **(D)**; and quantitative standardized data for comparing the fold changes in fl-APP mRNA expression as measured using RT-PCR **(E)**. Cells were treated with vehicle (0.1% DMSO), apoE4 (7.5 μg/mL), or drug control (100 μM fenofibrate, 6.25 μM naringenin, and 100 μM diosmetin) for 24 h. Pretreatment consisted of fenofibrate, naringenin, and diosmetin at different doses (see Figure 1A) for 1 h prior to stimulation with apoE4 for 24 h. The top panels in the figure part labels **A**, **B**, and **C** are representative blots, and their corresponding bottom diagrams are the quantitative evaluation of blots for the expression of fl-APP normalized with their respective β-actin levels in cells. For statistical evaluations, the two-way ANOVA analysis followed by Tukey’s range test was performed. Statistically significant differences (#*p* < 0.05) compared with control; (*p < 0.05) compared with apoE4 treated cells only; not significantly different (ns) compared to control cells. Data are expressed as percentage of protein expression compared with control, and mean ± S.D. of at least three separate experiments.

B103-hAPP695wt cells control was considered as 100%. ApoE4 treatment has significantly increased the expression of fl-APP by 1.13-, 1.21-, and 1.18-folds in fenofibrate, naringenin, and diosmetin experiments, respectively, as compared to control cells (Figure 2D). The expression of fl-APP was not significantly different in vehicle and drug control (fenofibrate, naringenin, and diosmetin only) as compared to control cells. These results showed that neither DMSO nor drugs alone affect fl-APP expression (Figure 2D).

Pretreatment of cells with 25, 50, and 100 μM of fenofibrate (Figure 2A); and 6.25, 12.5, and 25 μM of naringenin (Figure 2B) showed no significant effect on the fl-APP expression level as compared to apoE4 treated cells. However, pretreatment of cells with 25, 50, and 100 μM of diosmetin (Figure 2C) significantly and dose-dependently decreased the fl-APP expression level by 0.17-, 0.63-, and 0.99-folds, respectively, as compared to apoE4-treated cells.

Similar levels of fl-APP expression were obtained with both APP mAb 3E9 (Figures 2A–C) and β- Amyloid (6E10) Ab (Figure 2D) antibodies confirming that the signals correspond to the levels of fl-APP rather than APP-like proteins (APLP1 and APLP2). The lack of significant differences in the corresponding β-actin immunoreactivity among the groups suggests that differences in the expression of fl-APP are not attributed to loading different amounts of proteins per lane.

These results indicate that apoE4 increases the expression of fl-APP which may then be used as a substrate for β-secretase followed by γ-secretase cleavage leading to increased Aβ production. While pretreatment with fenofibrate and naringenin did not prevent the increase in apoE4-induced fl-APP expression, diosmetin dose-dependently decreased the apoE4-induced fl-APP expression. These data indicate that fenofibrate and naringenin may both decrease Aβ protein levels by a distinct mechanism from diosmetin.

The mRNA and protein levels are often correlated. The increase in the APP protein expression may be due to the apoE4-stimulated upregulation of APP transgene expression or APP mRNA stabilization. To test this hypothesis, we performed quantitative reverse transcription-PCR using APP-specific primers to amplify RNA isolated from B103-hAPP695wt control and treated cells. The treatment of cells with vehicle, drug control (fenofibrate, naringenin, and diosmetin alone), or pretreatment with fenofibrate and flavonoids had no effect on the APP mRNA expression in the B103-hAPP695wt cells as compared to the apoE4 treated cells (Figure 2E). β-Actin was used as a reference gene for the calculation of fold changes in APP mRNA expression using the delta–delta CT method. These results demonstrate that the increase in the APP protein expression was not due to an effect of apoE4 on the APP transgene expression or mRNA stability but may be rather due to post-translational processing of APP. This result is corroborated by the Tasaki group which looked at the differential expression of AD genome-wide association study (GWAS) genes at the protein and the mRNA level and showed that the APP protein exhibited significantly higher association with cognition than its mRNA in AD (Tasaki et al., 2022).

### 3.3 Effect of fenofibrate and flavonoids on apoE4 – induced sAPP production

To understand how fenofibrate and flavonoids reduce the apoE4-induced Aβ production, we next investigated their effects on the apoE4-induced APP processing. Intracellular fl-APP is processed by either α- or β-secretase enzymes releasing the N-terminal soluble APP derivatives sAPPα and sAPPβ, respectively, in the conditioned medium. To determine the relative amount of sAPPtotal (sAPPα and sAPPβ) and sAPPα released into the conditioned medium, media were harvested, and proteins were probed with two different antibodies (human APP mAb 3E9 and 6E10 mAb). The APP mAb 3E9 is specific against the N-terminus of APP and cannot distinguish between various sAPP isoforms, while the 6E10 Ab is specific against sAPPα resulting from α-secretase cleavage but not β-secretase cleavage.

**Figure 3:**
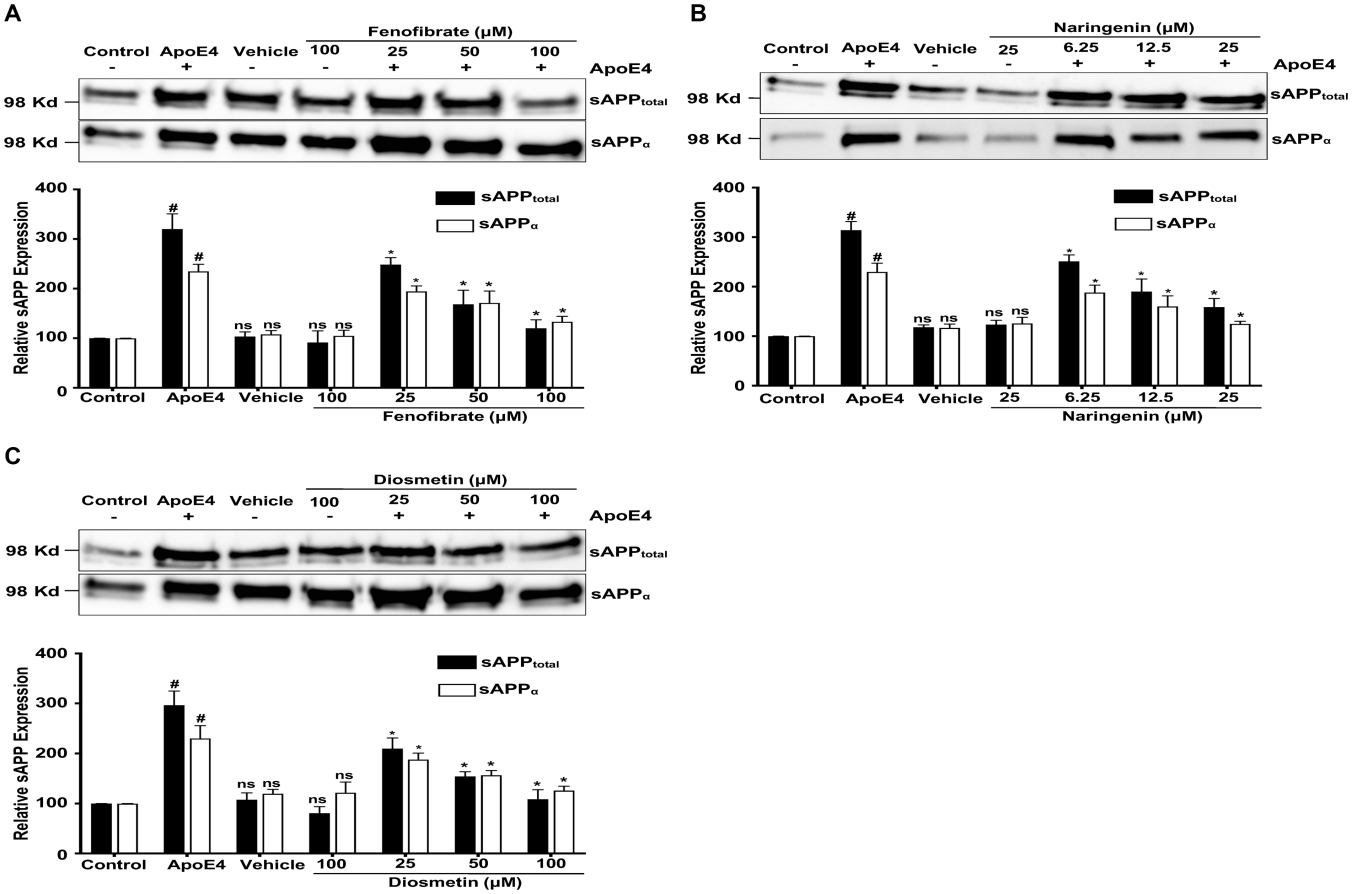
Effect of fenofibrate **(A)**, naringenin **(B)**, and diosmetin **(C)** on the apoE4-induced secretion of sAPPtotal and sAPPα in the conditioned medium. Cells were treated with vehicle (0.1% DMSO), apoE4 (7.5 μg/mL), or drug control (100 μM fenofibrate, 6.25 μM naringenin, and 100 μM diosmetin) for 24 h. Pretreatment consisted of fenofibrate, naringenin, and diosmetin at different doses (see Figure 1A) for 1 h prior to stimulation with apoE4 for 24 h. The top panels in the figure part labels **A**, **B**, and **C** are representative blots, and their corresponding bottom diagrams are the quantitative evaluation of blots for the expression of sAPPtotal and sAPPα normalized with their respective β-actin levels in cells. For statistical evaluations, the two-way ANOVA analysis followed by Tukey’s range test was performed. Statistically significant differences (#*p* < 0.05) compared with control; (*p < 0.05) compared with apoE4 treated cells only; not significantly different (ns) compared to control cells. Data are expressed as a percentage of protein expression compared with control and mean ± S.D. of at least three separate experiments.

Figure 3 represents the effects of fenofibrate, naringenin, and diosmetin on apoE4-induced sAPPtotal (sAPPα and sAPPβ) and sAPPα secretion in B103-hAPP695wt cells. The apoE4 treatment has significantly increased the secretion of sAPPtotal by 2.20-, 2.14-, and 1.96-folds and of sAPPα by 1.34-, 1.29-, and 1.30-folds in fenofibrate, naringenin, and diosmetin conditions, respectively, as compared to control cells. There were no significant changes in the secretion of sAPPtotal and sAPPα observed in cells treated with vehicle and drug control as compared to control cells. However, pretreatment of cells with 25, 50, and 100 μM fenofibrate has significantly reduced the secretion of sAPPtotal by 0.71-, 1.51-, and 2.00-folds and sAPPα by 0.40-, 0.63-, and 1.01 folds, as compared to apoE4 treated cells (Figure 3A). Similarly, pretreatment of cells with 6.25, 12.5, and 25 μM naringenin decreased the secretion of sAPPtotal by 0.63-, 1.24-, and 1.55-folds and sAPPα by 0.41-, 0.69-, and 1.05-folds (Figure 3B). In total, 25, 50, and 100 μM doses of diosmetin decreased the secretion of sAPPtotal by 0.86-, 1.42-, and 1.87-folds and sAPPα by 0.42-, 0.74-, and 1.04-folds, respectively, as compared to apoE4 treated cells (Figure 3C). The immunoblots for secretion of sAPPtotal and sAPPα were normalized with respective cellular protein concentrations in treated B103-hAPP695wt cells.

Diosmetin exhibited the most significant impact on sAPPtotal secretion, followed by fenofibrate and then naringenin. In contrast, the secretion of sAPPα was similarly decreased to comparable levels by diosmetin, naringenin, and fenofibrate. In addition, at the highest doses, diosmetin and fenofibrate both decreased sAPPtotal and sAPPα secretions to the same levels (Figures 3A,C), thus indicating their interference with β-secretase cleavage. Naringenin, on the other hand, at the highest doses has less effect on sAPPtotal secretion indicating that interference with β- secretase cleavage may not be the only mechanism by which it lowers Aβ production. This finding clearly demonstrates that all treatments prevented the apoE4-induced APP processing while interfering with secretases.

### 3.4 Effect of fenofibrate, naringenin, and diosmetin on apoE4-induced CTFα and CTFβ production

As reported earlier, α- and β-secretases cleave the APP to generate membrane-bound CTFα and CTFβ, respectively. The CTFα and CTFβ serve as substrates for γ-secretase to generate the P3 fragment and Aβ peptide, respectively. Studies have reported that the inhibition of γ-secretase results in the accumulation of CTFs and the reduction in Aβ generation (Mitani et al., 2012). To determine at which level fenofibrate and flavonoids affect apoE4-induced APP processing, we measured the expression of CTFα and CTFβ.

**Figure 4:**
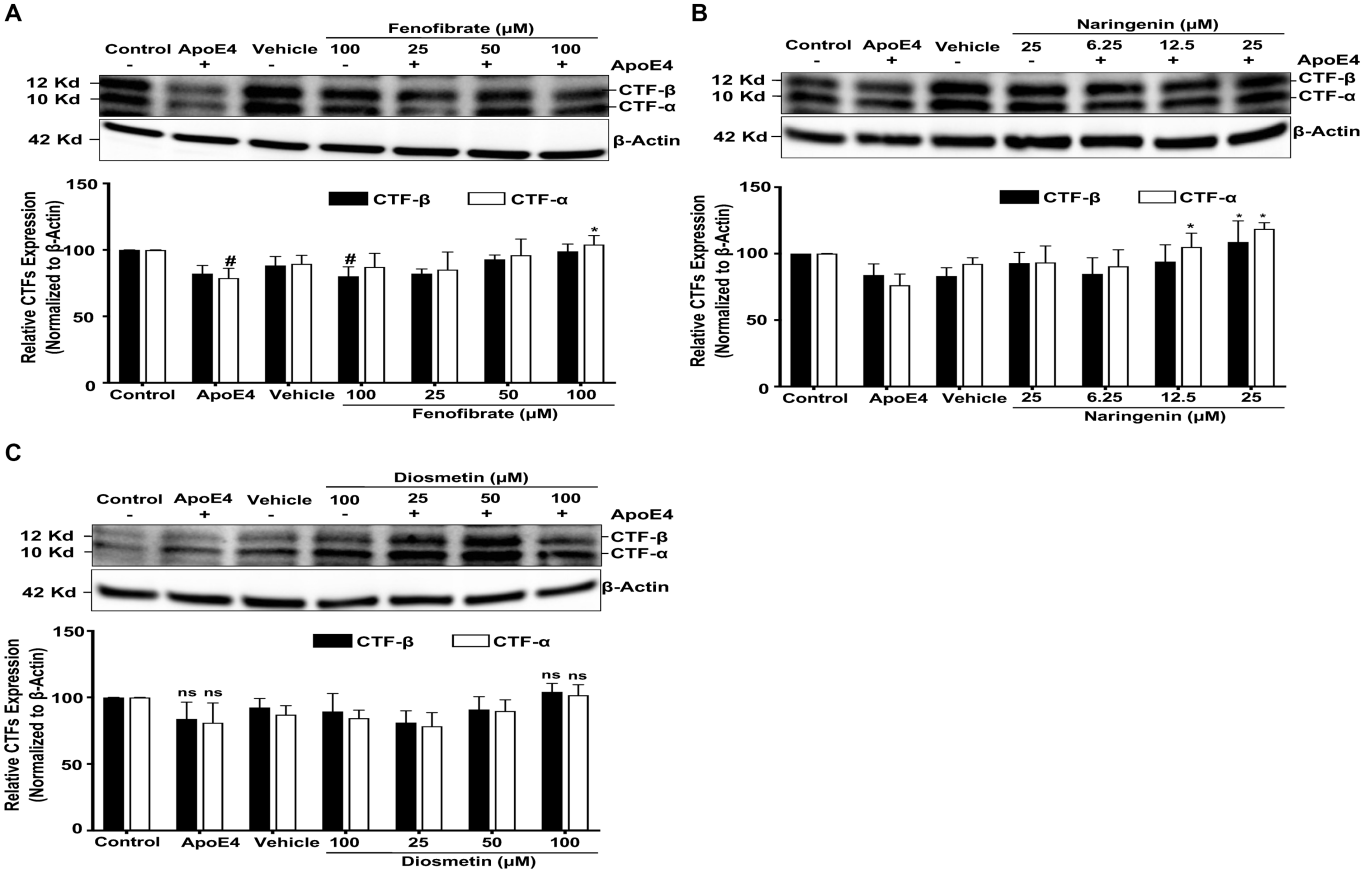
Effect of fenofibrate **(A)**, naringenin **(B)**, and diosmetin **(C)** on the apoE4-induced CTF–α and –β. Cells were treated with vehicle (0.1% DMSO), apoE4 (7.5 μg/mL), or drug control (100 μM fenofibrate, 6.25 μM naringenin, and 100 μM diosmetin) for 24 h. Pretreatment consisted of fenofibrate, naringenin, and diosmetin at different doses (see Figure 1A) for 1 h prior to stimulation with apoE4 for 24 h. The top panels in the figure part labels **A**, **B**, and **C** are representative blots, and their corresponding bottom diagrams are the quantitative evaluation of blots for the expression of CTFα and CTFβ normalized with their respective β-actin levels in cells. For statistical evaluations, the two-way ANOVA analysis followed by Tukey’s range test was performed. Statistically significant differences (#*p* < 0.05) compared with control; (*p < 0.05) compared with apoE4 treated cells only; significantly different (ns) compared to control cells. Data are expressed as percentage of protein expression compared with control, and mean ± S.D. of at least three separate experiments.

Cells treated with apoE4 caused a decrease in the production of CTFα and CTFβ, as compared to control cells. This decrease in the level of CTFα and CTFβ level may be due to apoE4-induced processing by γ-secretase of CTFs to Aβ peptides. Cells pretreated with fenofibrate (Figure 4A) and naringenin (Figure 4B) caused a dose-dependent accumulation of apoE4-induced CTFα and CTFβ that was significant with the highest doses, especially with CTFα, while diosmetin (Figure 4C) showed no significant accumulation in the CTFα and CTFβ levels. Taken together, these data clearly indicate that apoE4 has increased the processing and/or transport of APP resulting in an increased production of Aβ which was inhibited by fenofibrate and flavonoids.

## 4 Discussion

AD is a multifaceted disorder influenced by genetic and environmental risk factors. The E4 allele of the human *ApoE* gene constitutes a high-risk factor for LOAD (Kim et al., 2009). People who are homozygous for this allele have elevated serum cholesterol levels and approximately 10 times greater risk for AD (Yu et al., 2014; Cuyvers and Sleegers, 2016; Sims et al., 2020). ApoE4 also plays a relevant role in cholesterol transport and metabolism in healthy brains (Burns and Rebeck, 2010; Leduc et al., 2010; Flowers and Rebeck, 2020). In addition, studies reported that the cholesterol-rich lipid rafts of cell membranes host the β- and γ-secretases, indicating their activities might be affected by the levels of cholesterol in cell membranes (Xiong et al., 2008). Furthermore, large-scale GWA studies on AD not only confirmed that apoE4 is a major risk factor but also provided evidence that other genetic susceptibility loci include proteins involved in lipid metabolism (Kunkle et al., 2019). This suggests that the effect of apoE4 on Aβ production may serve as a potential target for disease-modifying treatment (Hunsberger et al., 2019). Others have reported that cholesterol-modulating agents may be a promising treatment for AD (Golde and Eckman, 2001).

This study evaluated the effects of cholesterol modifying agents–fenofibrate, naringenin, and diosmetin, on apoE4-induced APP processing into Aβ peptides using B103 neuroblastoma cells stably transfected with wild-type human APP695. Aβ is produced by the sequential proteolytic cleavage of APP. The first cleavage by β-secretase (BACE1) generates the CTFβ fragment (otherwise known as C99), and the second cleavage is induced by γ-secretase to generate the Aβ peptide (Vassar et al., 1999). Our data shows that apoE4 has induced extracellular Aβ secretion and intracellular Aβ production, and this is consistent with results from prior studies (Ye et al., 2005). The apoE4-induced Aβ secretion and production was significantly reduced with fenofibrate, naringenin, and diosmetin, supporting the hypothesis that cholesterol-modulating agents might affect apoE4-induced APP processing.

To elucidate the Aβ reducing property of these agents, we measured the expression of Aβ precursor, fl-APP. ApoE4 treatment has significantly increased the expression of fl-APP indicating that higher APP levels served as substrate for β- followed by γ-secretase cleavage leading to the increase in Aβ production. While fenofibrate and naringenin did not reduce the apoE4-induced fl-APP expression, diosmetin has dose-dependently inhibited the apoE4-induced fl-APP expression. The increase in intracellular fl-APP was not due to the effect of apoE4 on APP transgene expression or mRNA stability, as confirmed by the qRT-PCR experiment. This indicates that apoE4 affected post-translational processing of APP (Wolozin et al., 1996).

The APP protein trafficking between the secretory and endocytic pathways involves post-translational modifications such as phosphorylation at the c-terminal domain (Haass et al., 2012; Wang et al., 2017). While phosphorylation of serine 655 (Ser655) at the c-terminal APP decreases α-secretase action (Gandy et al., 1988; Isohara et al., 1999), phosphorylation of threonine 668 (Thr 668) increases APP and β-secretase (BACE1) colocalization, thus promoting β-secretase cleavage and increasing Aβ levels (Lee et al., 2003). Thr 668 is phosphorylated by several kinases including glycogen synthase kinase 3β (GSK3β) (Aplin et al., 1996). This can lead to APP conformational changes affecting its interaction with secretases (Ramelot and Nicholson, 2001). Indeed, a study showed that diosmetin inhibited both GSK-3α and GSK-3β. The inhibition of GSK-3α activity prevented the interaction between APP and γ-secretase, and the inactivation of GSK-3β prevented the phosphorylation of Thr 688 and thus blocked APP- β-secretase (BACE1) interaction, interrupting the amyloidogenic pathway (Sawmiller et al., 2016). This confirms our finding that diosmetin may have decreased apoE4-induced Aβ production by preventing post-translational modification such as inhibiting GSK-3β phosphorylation of APP at Thr 688, interfering with the β-secretase (BACE1) action, and/or inhibiting GSK-3α, and thus preventing the interaction of y-secretase with CTFβ. Alternatively and as reported by other studies, enhanced cleavage of CTFβ by α-secretase to less toxic Aβ could also be a possible explanation when the γ-cleavage pathway is inhibited (Beher et al., 2002; Cook et al., 2010; Portelius et al., 2011). Others showed that apoE4 stimulates *APP* gene transcription via the c-fos signaling pathway, leading to increased levels of Aβ (Huang et al., 2017), while another study reported that apoE4 increased β-secretase (BACE1) and Aβ levels, independently of cholesterol efflux (Dafnis et al., 2018). These observations indicate that apoE4 may play a dual role as a lipid transport and signaling molecule in the brain.

The N-terminal soluble APP derivatives can be released in the conditioned medium by either α- or β-secretase cleavage of fl-APP. The secretion of sAPPtotal in the conditioned medium reflects the effects of both α- and β-secretase on fl-APP processing, while sAPPα indicates only the effect of α-secretase cleavage. In this study, the increased secretion of both sAPPtotal and sAPPα with apoE4 suggests increased processing of fl-APP via both α- and β-secretases. On the other hand, all treatments dose-dependently decreased the apoE4-induced α- and β-secretase cleavages. At the highest doses, diosmetin and fenofibrate had more pronounced lowering effects on sAPPtotal as compared to sAPPα level, thus indicating their interference with β-secretase cleavage while promoting α-cleavage of APP. In fact, α-secretase processes 90% of total APP, while β-secretase cleaves by 10% (Hardy and Selkoe, 2002). This is consistent with our results showing increased α-secretase activity relative to β cleavage when comparing the levels of sAPPtotal to sAPPα at high doses. Naringenin, on the other hand, at the highest doses, has less effect on sAPPtotal secretion indicating that interference with β- secretase cleavage may not be the only mechanism by which it lowers Aβ production.

The processing of fl-APP via α- and β- secretases also generates membrane-bound CTFα and CTFβ, respectively. These CTFα and CTFβ are the substrate for the γ-secretase, leading to the production of p3 fragments and Aβ, respectively. The accumulation of CTFs in the cell reflects the inhibition of γ-secretase activity (Citron, 2004). The reduced levels of both CTFα and CTFβ with apoE4 treatment indicate that γ-secretase processing of CTFs is increased leading to increased Aβ production as shown in our study. On the other hand, Aβ reduction with fenofibrate and naringenin was accompanied by a dose-dependent accumulation of both CTFα and CTFβ, while diosmetin showed a potent ability to reduce Aβ levels without a significant increase in CTFs. Thus, accumulation of CTFβ and CTFα after drug treatment could reflect a possible inhibition of γ-cleavage or stimulation of α-cleavage by fenofibrate and naringenin. This supports our results where both fenofibrate and naringenin did not prevent apoE-4 post-translational modifications but most likely reduced Aβ by either inhibiting β-secretase (BACE-1) and/or stimulating α secretase enzymes. Indeed, a study found that fenofibrate increased the expression of PPAR-α, decreased β-secretase (BACE-1) mRNA and protein levels, and reduced soluble APPβ (sAPPβ) and Aβ42 release, but it did not modify the levels of APP and presenilin 1 (PS1) (Zhang et al., 2014). Another study showed that PPARα agonists activate α-cleavage of APP (Corbett et al., 2015) but inhibit β-secretase enzyme with no effect on the level of APP and Presenilin-1 (PS1) (Zhang et al., 2015). Our study evaluated the effects of fenofibrate (25–100 μM) on apoE4-induced Aβ production. The findings with fl-APP, sAPPβ, and Aβ production are supported by the study of Zhang et al. (2014) and Zhang et al. (2015).

Several studies have investigated the Aβ-lowering effects of γ-secretase inhibitors (GSIs) and modulators (GSMs) (Kreft et al., 2009; Oehlrich et al., 2011). GSIs reduce the dendritic spine density in normal mice but not in APP-knock-out mice, suggesting that the accumulation of either CTFα or β or both may cause synaptic toxicity and a potential cause of memory impairment (Dewachter et al., 2002; Bittner et al., 2009). In contrast, GSMs reduce Aβ peptides without increasing CTFβ (Mitani et al., 2012). Our results show that while naringenin and fenofibrate may behave as GSIs, diosmetin behaves more as GSM by not increasing the CTFβ levels.

Naringenin has been widely studied for its neuroprotective action as well as for its ACAT inhibitory activity in the *in vivo* and *in vitro* models. Evidence indicates that the distribution of cholesterol in the form of free cholesterol (FC) and cholesteryl esters (CE) within cells regulates Aβ generation (Puglielli et al., 2001; Huttunen and Kovacs, 2008). The balance between FC and CE levels is maintained by an endoplasmic reticulum (ER)-resident enzyme, ACAT. A study reported that the inhibition of ACAT activity potently reduced Aβ generation and both CTF α and CTFβ fragment levels (Puglielli et al., 2001), while another study showed that ACAT activity could regulate APP trafficking in the early secretory pathway and consequently the availability of APP for Aβ generation (Huttunen et al., 2009). Furthermore, it has been demonstrated that naringenin effectively mitigates Aβ-induced neuronal toxicity in PC12 cells. This effect is achieved by enhancing the phosphorylation of GSK-3β, which, in turn, inhibits it and subsequently diminishes the amyloidogenic pathway (Zhang et al., 2018). In addition, nano-emulsions of naringenin downregulated APP and β-secretase (BACE1) expression in Aβ-induced neurotoxicity in a human neuroblastoma cell line (SH-SY5Y) (Md et al., 2018). Our study showed that naringenin decreased sAPPtotal over sAPPα most likely by interfering with β-secretase (BACE1) activity and also promoted α-cleavage by increasing the CTF α over CTFβ levels. This is the first study to investigate naringenin’s effect on apoE4-induced APP processing, which results in the reduction of Aβ production. This effect may be attributed to both the inhibition of ACAT and the modulation of APP trafficking and processing into Aβ.

In summary, the tested agents mentioned above showed a potential to limit Aβ generation. Knowing that the expression of PPARs is modified in the AD brain, the characterization of new agents able to activate several PPARs isoforms could be needed for an efficient treatment for AD (Sáez-Orellana et al., 2020; Wójtowicz et al., 2020). Since neither cure nor treatment to alter the progression of AD has been observed until now (Athar et al., 2021), alternative strategies could be therefore to develop agonists that can simultaneously activate PPARα and PPARγ such as fenofibrate and diosmetin and/or inhibit ACAT activity such as naringenin. Indeed, lactoferrin-modified long-circulating liposomes for brain-targeted delivery of diosmetin have been developed as a potential therapy for AD patients (Sun et al., 2022). Future research will aim to investigate the specific molecular mechanisms of fenofibrate, naringenin, and diosmetin in reversing apoE4-induced Aβ upregulation as well as the direct involvement of the PPARs and ACAT in reversing apoE4 effects on Aβ production.

## Data availability statement

The raw data supporting the conclusions of this article will be made available by the authors, without undue reservation.

## Author contributions

VD: conception, planning, performed the experiments, analyzed data, prepared first draft. KB: conception and design, wrote the manuscript, supervision, project administration, resources. All authors contributed to the article and approved the submitted version.
